# Assessment of serum arginase I as a type 2 diabetes mellitus diagnosis biomarker in patients

**DOI:** 10.3892/etm.2014.1768

**Published:** 2014-06-06

**Authors:** SONG WANG, FANG FANG, WEN-BO JIN, XIA WANG, DA-WEI ZHENG

**Affiliations:** 1 Department of Endocrinology, Henan Nanyang Central Hospital, Nanyang, Henan 473000, P.R. China; 2 Department of Computed Tomography, Henan Nanyang Central Hospital, Nanyang, Henan 473000, P.R. China; 3 Department of Nursing, Henan Nanyang Central Hospital, Nanyang, Henan 473000, P.R. China; 4 Department of Respiratory Medicine, Henan Nanyang Central Hospital, Nanyang, Henan 473000, P.R. China

**Keywords:** diabetes mellitus, arginase I, biomarker, diagnosis

## Abstract

Previous studies have reported that levels of serum arginase I are increased in certain diseases. However, the exact association between arginase I and diabetes mellitus (DM) has yet to be determined. The aim of the present study was to investigate the correlation between arginase I activity and DM to determine whether arginase I activity may be used as a diagnostic biomarker for DM. DM was induced by a streptozotocin injection, while the arginase inhibitor, citrulline, was administered daily. Serum levels of glucose, reactive oxygen species (ROS) and arginase I activity were analyzed, and quantitative polymerase chain reaction and western blot analysis were performed to detect the mRNA and protein expression levels of arginase I, respectively. In addition, western blot analysis was used to determine the protein expression of the Tie 2 receptor. Pearson’s analysis was used to determine the correlation between arginase I activity and Tie 2 expression, while concordance analysis was performed using the Cohen’s test to obtain the Kappa statistic. The results demonstrated that serum arginase I activity levels in the rats with DM were significantly elevated compared with the control group, and positively correlated with the blood glucose levels. In addition, the blood glucose and ROS levels were increased significantly in the rats with DM. Arginase I mRNA and protein expression levels were significantly elevated in the diabetic rats when compared with the control group, and Tie 2 expression levels increased and were shown to correlate with arginase I activity in the diabetic rats. In addition, arginase I activity was shown to correlate with glucose intolerance and post-load glucose values. Good concordance was observed between arginase I activity and the clinical diagnosis for DM (κ=0.876; P<0.001). Therefore, the results indicated that arginase I may function as a diagnostic biomarker for DM rats model.

## Introduction

Arginase enzymes play a major role in the biosynthesis of polyamines and amino acids, including ornithine, proline and glutamate (1,2). Arginase has two distinct isoforms, arginase I and II, which differ in subcellular localization. Arginase I is predominantly localized in the cytosol of hepatic cells and is a key enzyme in the urea cycle. Arginase II is expressed in the mitochondria of extrahepatic cells and is encoded by a different gene (3). The two arginases are constitutively expressed in cells and tissues, and indirectly regulate nitric oxide (NO) generation from nitric oxide synthase (NOS) by competition for a common enzyme substrate (4,5). Therefore, the induction of NOS arginase has been investigated in the inflammatory cells of asthmatic lungs as pathophysiological evidence that the consumption of L-arginine by arginase may lead to the depletion of NO production and endothelial dysfunction; thus, the enlargement of bronchial smooth muscle associated with airway hyperresponsiveness (6–8). Furthermore, following catabolism by arginase, arginine is no longer available to NOS; thus, subsequent NO synthesis is diminished (9).

Diabetes mellitus (DM) is a worldwide disease that is frequently associated with a high risk of atherosclerosis and renal, nervous system and ocular damage (10). Oxidative damage is involved in DM and the associated complications (11), with reactive oxygen species (ROS) being implicated in the pathogenesis of DM (12). Patients with type 2 DM frequently exhibit vascular endothelium dysfunction associated with hypercholesterolemia, and NO deficiency is a major factor contributing to endothelial dysfunction, which has been demonstrated in hypertension, tobacco smoking and malaria (13). Furthermore, an increased production of ROS has been shown to be associated with protein glycation and/or glucose auto-oxidation in patients with DM (14).

Serum arginase levels have been analyzed in a number of diseases using activity assays and enzyme-linked immunosorbent assay (ELISA) methods. For patients with DM, although serum arginase activity levels remain controversial (15), serum arginase I levels have been shown to be elevated using ELISA (16). In other diseases, including sickle cell disease (17), retinopathy (18) and cardiovascular disease (19), changes in arginase I levels have also been observed. However, little is known with regard to the expression levels of arginase I in patients with type 2 DM, and the association with DM remains elusive.

Therefore, in the present study, the serum levels of arginase I in patients with DM were determined. In addition, the use of arginase I as a diagnostic biomarker for type 2 DM was investigated.

## Material and methods

### Animals and model establishment

All experiments were performed in accordance with the institutional animal care and use guidelines of the Ethical Committee of the Henan Nanyang Central Hospital (Nanyang, China). Male Wistar rats (age, ~10 weeks; weight, ~200 g) were used in the study and housed in standard light/dark cycles, with access to a standard rat diet and water *ad libitum*.

The animals were randomly divided into three groups, including control, diabetic model and citrulline-treated diabetic groups. DM was induced by a single intraperitoneal injection of 50 mg/kg streptozotocin (STZ; (Beijing, China) and treated with citrulline in distilled water by orogastric gavage for six weeks of study, while the control and diabetic groups received water as a vehicle. The doses of citrulline were selected based on the reported arginase inhibiting activity (20) and confirmed by arginase activity measurement.

### Serum blood collection

The rats were fully anesthetized with 3% pentobarbital sodium, and the chest was opened. A needle was inserted through the diaphragm and into the heart. Negative pressure was gently applied once the heart had been punctured, and the needle was repositioned as required until blood flowed into the syringe. The blood collected from the rats was allowed to clot at room temperature. The serum was separated by centrifugation at 5,000 g for 10 min, and aliquots of serum were stored at −80°C until required for further analysis.

### Arginase I activity

Arginase activity was analyzed as previously described (21). Briefly, 100 μl serum was incubated with equal volumes of 10 mM manganese chloride in 50 mM Tris-HCl (pH 7.4) (Beijing, China) at 55°C for 10 min to activate the enzyme. All the experimental procedures were performed according to the methods previously described by Giri *et al* (22). The samples were then transferred to a 96-well plate for ELISA analysis at 540 nm.

### Quantitative polymerase chain reaction (PCR)

Total RNA was isolated from the collected blood using TRIzol reagent (Sigma-Aldrich, Beijing, China), according to the manufacturer’s instructions. Following DNAse treatment, total RNA (1 μg) was reverse transcribed using Moloney Murine Leukemia Virus Reverse Transriptase and oligo(dT) 12–18 primers. A reaction performed in the absence of the reverse transcriptase enzyme functioned as a negative control. Amplified cDNA was subjected to PCR using specific gene primers that are listed in Table I, according to the methods previously described by Giri *et al* (22). GADPH was used as an internal control.

### Western blot analysis

All lysates extracted from the blood were separated by 15% SDS-PAGE and electrotransferred onto nitrocellulose membranes. The membranes were blocked with 5% defatted milk in phosphate-buffered saline (PBS) overnight at 4°C, and then incubated with monoclonal antibodies against arginase I (1:2,000), mouse anti-Tie 2 (1:3,000) and anti-human β-actin (1:1,000; Santa Cruz Biotechnology, Inc., Santa Cruz, CA, USA) for 2 h at room temperature. Next, the membranes were incubated with a horseradish peroxidase-conjugated anti-mouse (1:3,000) secondary antibody (Santa Cruz Biotechnology). Reactive signals were visualized using an enhanced chemiluminescence kit (PE Applied Biosystems, Foster City, CA, USA).

### Statistical analysis

All data are presented as the mean ± standard deviation. Mean values were compared between groups using the unpaired Student’s t-test for normally distributed variables, while Pearson’s analysis was computed to determine the correlations between variables. P<0.05 was considered to indicate a statistically significant difference. If variables were not normally distributed, data were log-transformed prior to correlation analysis. Concordance analysis was performed using Cohen’s test.

## Results

### Blood and serum parameters

**Figure 1:**
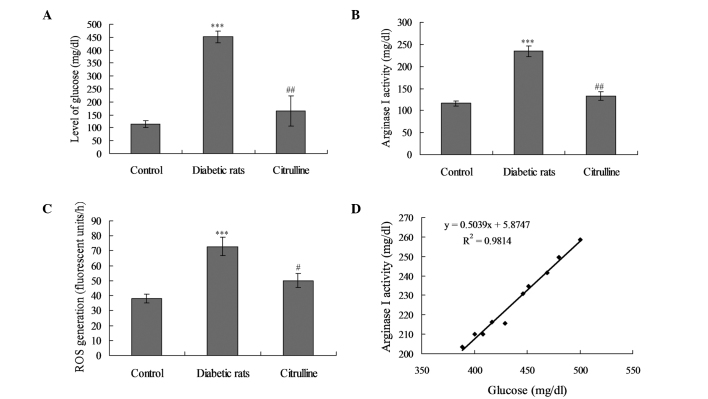
Effect of STZ-induced DM and the administration of citrulline on (A) blood glucose levels, (B) arginase I activity, (C) ROS generation and (D) correlation between arginase I activity and the level of glucose. ^***^P<0.001, vs. control rats; ^##^P<0.01 and ^#^P<0.05, vs. control rats. ROS, reactive oxygen species; STZ, streptozotocin; DM, diabetes mellitus.

Treatment with 50 mg/kg STZ triggered a significant increase in the blood glucose levels when compared with the control rats (Fig. 1A; P<0.001). However, arginase inhibition by citrulline did not exhibit any significant effect on the glucose levels.

Serum arginase I activity levels in diabetic rats treated with STZ were significantly elevated when compared with the control group (Fig. 1B; P<0.001). However, activation of arginase I was significantly inhibited following the administration of an arginase inhibitor (P<0.05). With regard to ROS generation, ROS levels were significantly increased in the diabetic rats when compared with the control and citrullin-treated rats (Fig. 1C; P<0.001). Furthermore, arginase I activity levels significantly correlated with the blood glucose levels in the diabetic rats (Fig. 1D; P<0.01, r=0.8672), but not in the arginase inhibitor group (data not shown).

### mRNA and protein expression levels of arginase I are enhanced in diabetic rats

**Figure 2:**
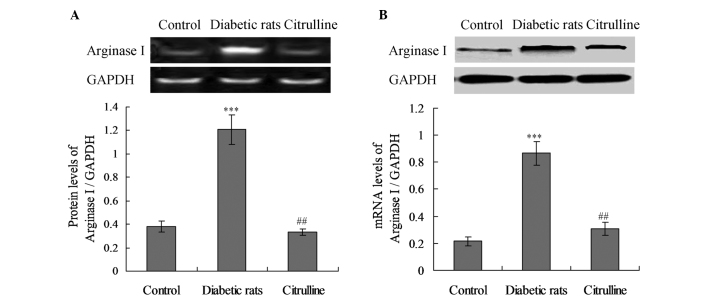
(A) Protein and (B) mRNA expression levels of arginase I in the diabetic rats. ^***^P<0.001, vs. control rats; ^##^P<0.01, vs. diabetic rats.

To further evaluate the effect of STZ-induced DM on the circulating levels of arginase I, the mRNA and protein expression levels of arginase I were analyzed in the blood serum. As shown in Fig. 2, no statistically significant difference in the mRNA expression levels of arginase I was observed between the control and citrulline groups (Fig. 2B; P>0.05). However, the mRNA expression levels of arginase I in the diabetic rats were significantly increased compared with the control group (Fig. 2B; P<0.001). Similarly, the arginase I protein expression levels were enhanced in the diabetic rats when compared with the control rats (Fig. 2A; P<0.001).

### Expression levels of Tie 2 are increased and correlate with arginase I activity in diabetic rats

**Figure 3:**
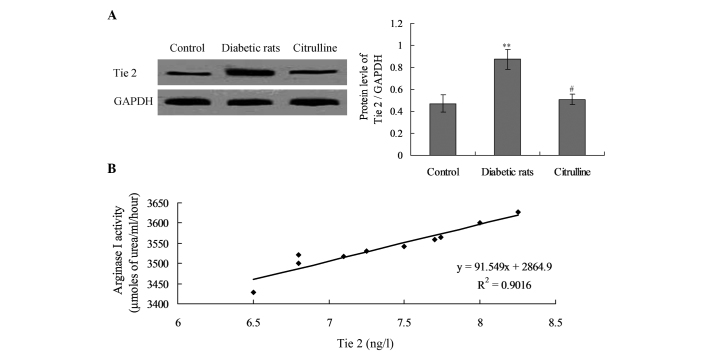
Tie 2 expression levels were enhanced and correlated with arginase I activity in the diabetic rats. (A) Expression and statistical analysis of the Tie 2 receptor. (B) Correlation analysis between arginase I activity and Tie 2 expression levels in the diabetic rats. ^**^P<0.01, vs. control rats; ^#^P<0.05, vs. diabetic rats.

In order to investigate the cause of arginase I activity, the expression levels of the arginase I-associated receptor, Tie 2, were detected in diabetic rats. The results indicated that Tie 2 expression in diabetic rats was significantly increased compared with the control group (Fig. 3; P<0.01). However, citrulline treatment was found to significantly decrease Tie 2 expression in the diabetic rats (Fig. 3; P<0.05).

Furthermore, the correlation between arginase I activity and Tie 2 expression levels was analyzed by correlation analysis. The results revealed that arginase I activity positively correlated with Tie 2 receptor expression in the diabetic rats (Fig. 3B; P<0.01, r=0.9013).

### Arginase I activity reflects glucose intolerance and post-load glucose levels

In order to investigate the associations between arginase I activity and other blood parameters, Spearman’s correlation analysis of arginase I activity with other blood parameters was performed. As shown in Table II, arginase I activity exhibited a significant positive correlation with glucose intolerance and post-load glucose values in the diabetic rats.

### Concordance analysis

Arginase I activity DM (+) and DM (−) characteristics were used to analyze the concordance with the clinical diagnosis of diabetic rats (Table III). The results demonstrated that eight diabetic rats (DM+) analyzed with arginase I activity were consistent with the clinical diagnosis, and the sensitivity was 80.0% (8/10). According to Kappa analysis, the concordance value between arginase I activity and the clinical diagnosis for DM was 0.876 (κ=0.876; P<0.001; Table III).

## Discussion

Recently, the potential role of arginase (including arginase I and II) in the pathogenesis of DM has been investigated (23,24). However, to the best of our knowledge, the present study is the first study investigating the potential role of arginase I as a diagnostic or prognostic marker for type 2 DM. In the present study, diabetic rats exhibited increased levels of arginase I, which correlated with the blood glucose level; thus, may contribute to the severity of DM in rats. Decreasing the arginase I activity in diabetic rats may potentially provide a therapeutic method for type 2 DM (23).

In the present study, the increased expression of ROS was found to be involved in the pathogenesis of DM in rats. The production of ROS has been attributed to protein glycation; thus, increased levels of ROS by-products may result in changes in energy metabolism and the antioxidant defense status, participating in vascular complications in patients with DM (25,26). Regulation of arginase activity in diabetic rats may aid ROS metabolism. ROS affect amino acid and cation transport through the blood cell membranes; for example, cystine transport when blood cells are exposed to oxidative stress. Therefore, adjusting arginase activity may be a potential therapeutic method for improving the symptoms of DM in rats. In the present study, citrulline was used to inhibit arginase I activity. The results demonstrated that the mRNA and protein expression levels of arginase I were increased in the diabetic rats when compared with control group, but not in the citrulline treatment group. This observation indicates that arginase I protein has an important role in the pathogenesis of DM, which is consistent with the results of previous studies (27,28).

In the present study, a significant increase in Tie 2 receptor expression and arginase activity levels was observed in the blood serum of the diabetic rats. The increase in Tie 2 expression in the blood serum may be due to the shredding of extracellular activity of matrix metalloproteinases (29,30), or due to the Golgi-mediated release of the stored pool of Tie 2 (31). In addition, the changes in arginase I expression levels may be associated with the changes in Tie 2 receptor expression in the diabetic rats. Although the exact effects of high expression levels of Tie 2 in the blood remain unclear, the effects are associated with arginase I activity.

Increased expression and activity levels of arginase I in the blood have been investigated in rats with type 2 DM, and inhibition of arginase I has been shown to exhibit a protective effect in DM rat models (32). The results of the present study indicated that the arginase I inhibitor, citrulline, significantly decreased the activity of arginase I, as well as the blood glucose levels of diabetic rats. These observations confirm that arginase I activity may reflect the severity of DM.

In order to evaluate the possible or potential role of arginase I as a prognostic marker for DM, concordance analysis was performed. The results indicated that the sensitivity for arginase I was 80.0% (8/10). According to Kappa analysis, the concordance value between arginase I activity and the clinical diagnosis of DM was 0.876 (κ=0.876; P<0.001). A κ-value of >0.75 is considered to exhibit a better concordance. Therefore, arginase I diagnostic analysis exhibited a better concordance with the clinical diagnosis of DM in the present study.

In conclusion, arginase I activity and expression levels were significantly higher in the diabetic rats when compared with the control rats, and were shown to positively correlate with the blood glucose levels of the control rats. These observations indicate that arginase I may be used as a prognostic or diagnostic marker for patients with DM. Thus, arginase I may be considered as a potential method to diagnose DM in clinical practice.

## Figures and Tables

**Table I:** Sequences of primers used for arginase I PCR.

Genes	Primer sequences	Product length (bp)
Arginase I	5′ ATGTCCCTAAGGGGCAGCCTCTCGCGT 3′ 5′ CACAGCTGTAGCCATCTGACACAGCTC 3′	340
GAPDH	5′ TGCCTCCTGCACCACCAACTGC 3′ 5′ AATGCCAGCCCCAGCGTCAAAG 3′	456

PCR, polymerase chain reaction; GAPDH, glyceraldehyde 3-phosphate dehydrogenase.

**Table II:** Spearman’s correlation analysis of arginase I activity with other parameters.

Parameters	Correlation coefficient (′)	P-value
Glucose intolerance	0.704	0.002
Post-load glucose	0.497	0.047
Fasting glucose	0.212	0.083
Angiopoietin 1	0.198	0.103
Angiopoietin 2	0.183	0.116

**Table III:** Concordance between arginase I activity and clinical diagnosis in diabetic rats.

	Arginase I activity		
		
Clinical diagnosis	DM (+)	DM (−)	Total
DM (+)	8	1	9
DM (−)	1	0	1
Total	9	1	10

The data represent the numbers of the rats with the specified characteristics. DM, diabetes mellitus.
